# A Short Account of Two Cases of Cancer of the Liver

**Published:** 1873-03-01

**Authors:** M. Reece

**Affiliations:** Abingdon, Ill.


### A SHORT ACCOUNT OF TWO CASES OF CANCER OF THE LIVER.

BY M. REECE, M.D., ABINGDON, ILL.

During the months of August, September, and October, it fell to my lot to be in attendance on two cases of cancer of the liver at the same time, a circumstance, I imagine, quite rare in the practice of physicians generally.

G. W., aged forty-eight; bilious temperament; farmer, had his attention arrested by a mole situated near the lower border of the ribs, on the left side. This mole was about half an inch in length, and a third of an inch in width. It was congenital. It frequently became sore from the friction of the clothing.

Fearing that it was, or might become a cancer, he wished to have it extirpated. He was told that it was not a cancer, and that he could have it removed at any time. Still, apprehensive that it was a malignant tumor, and with an undefined feeling of dread with regard to it, about the middle of last February he applied for its removal. It was destroyed by the application of caustic potash ; the sore caused by the potash healed perfectly.

About two months after the removal of the mole, a small tumor appeared in the left axilla. It was believed to be an enlarged axillary gland which would soon pass into suppuration. It however continued slowly to increase in size until it became as large as on orange. It was not very hard, but gave a doughy sensation to the touch. His general health during the early period of the growth of this tumor seemed to be good, but now gradually began to fail. Symptoms of dyspepsia, with severe pain in the stomach, came on. This pain increased in severity greatly. He referred all his pain and suffering to the region of the stomach. The stomach and bowels were greatly and constantly distended with gas.

About the last of August the pain in the stomach reached its greatest intensity. Previous to this time he had been able to walk around, but now was confined to his bed. There was but little pain in the tumor in the axilla.

He could only take the lightest nourishment, generally beef-tea with crackers. The legs and feet were edematous ; the urine was inky-black; the bowels constipated; the conjunctiva slightly tinged yellow; but no other symptoms of jaundice. Pulse 120 to 130. Temperature 102°. The tumor in the axilla continued to grow, the desire for food constantly diminished; and he gradually sunk, and died on the 4th of October.

*Post mortem* ten hours after death: No cadaveric rigidity. The body was not greatly emaciated. Considerable distension of the abdomen, especially in the region of the stomach. Just beneath the skin, over the chest, abdomen, and inner portion of the thighs, were numerous black nodules, varying from the size of a small shot to that of a hazel-nut. They were hard, and felt like a shot beneath the skin. When cut they creaked with the sound peculiar to scirrhus substances. Their material, in color, was of a sooty black.

Incising the tumor in the axilla, to the depth of two inches, a soft, pulpy substance was reached, of the consistence of syrup, and of a brownish-red color. The construction of this tumor was undoubtedly cancerous.

The stomach contained a pint and a half of a dirty, green fluid, and was distended with gas. With the exception of being unnaturally dilated, it was unaffected. The liver was of a black color, like black velvet. Over the upper and under surfaces were numerous spots about an inch in diameter. They were raised somewhat like a blister, and contained a black juice. Some were small, very much like a grape, with half its substance buried in the liver. Hanging from the under surface were three tumors of the size of a hulled walnut ; they were round, and attached to the liver by a small pedicle. They contained an inky-black substance. The posterior portions of the liver were attached to the wall of the chest, and to the diaphragm, by a cancerous mass as large as the two fists. There was a great deal of dyspnœa during life, and I suppose this to be the explanation of it. The gall-bladder was filled with bile, but was not diseased. The liver weighed seven pounds. The structure of the other organs was unaffected.

I suppose this to have been a case where the cancerous deposits were secondary, which, according to Frerichs, and other writers, is the most usual form of cancer of the liver; but the following I believe to have been a case of primary cancer of the liver.

Mrs. P., aged sixty, began to complain of a pain in her right side and shoulder during the month of August last. This gradually increased in intensity, and she also became deeply and universally jaundiced. The urine was of a saffron color, the fæces clay-colored. Her pulse varied from 80 to 90, the temperature of the body about normal. Her appetite disappeared, and her strength rapidly failed. Towards the last she vomited a bloody serum. She also passed the same material by the bowels. Ten days before her death, a hard mass, of the size of a hen's egg, could be felt beneath the border of the ribs, on the right side. A prominent feature in her case was a foreboding that she would not get well. This feeling existed before she was confined to her bed, or her disease was thought to be serious. This premonition also existed in the preceding case. It is a condition I have learned to respect as significant of grave disease. She died, from exhaustion, November 2d.

At the *post mortem*, the body did not appear much emaciated. The sub-cutaneous fat was in abundance. The liver at once revealed the cause of the jaundice. Its upper and under surfaces were studded with nodules, from the size of a quarter to that of a half-dollar—raised above the surface, depressed in the center, of a yellow color, and of a hard, cheesy consistence. These nodules were disseminated throughout the liver in great abundance; in the interior of its substance some being as large as an orange. There was indeed but very little of the healthy tissue of the liver remaining. The gall-bladder had disappeared. It seemed to have been surrounded by the cancerous material. A cavity formed, filled with gall-stones, varying in size from a small shot to a pigeon's egg. They were seventy-eight in number. One, as large as a hickory-nut, appeared to have ulcerated through the wall of the gall-bladder, and occupied a cavity by itself. The posterior portion of the right lobe of the liver was converted into a scirrhus mass as large as the fist. The other organs were healthy.

